# Committee on publication ethics

**DOI:** 10.4103/0976-500X.72371

**Published:** 2010

**Authors:** Jatinder Singh

**Affiliations:** *Section Editor, JPP*

http://publicationethics.org

The basic tenet of good publication practices is faith and confidence in the peer review process. In order to select the best quality of articles, the review and publication process has to be transparent, methodical and impartial. All stages of this process are explicitly built on ethical principles and informed decisions, most of which are based on the distinctive vision of the editors. The quality of research published in a journal depends in turn on the trust that researchers, authors and readers repose in the editorial policies of the journal. The influence of powerful financial and intellectual interests sometimes comes in the way of these policies. This makes it imperative to have clear guidelines and unambiguous policies in order to ensure the ethical treatment of all aspects of the editorial and publication processes.

What started out as an informal group of editors concerned at the increasing instances of authors flaunting scientific integrity evolved later into the Committee on Publication Ethics (COPE) in 1997. COPE is now a registered British charitable organization and has a number of leading journals and editors as its members. Editors of scientific journals are encouraged to submit issues relating to the integrity of the work submitted for publication and the website (http://publicationethics.org) acts as a forum for these wide-ranging discussions and their possible redressal. The summaries of all cases and their outcomes are posted on the website and act as a guide for authors and editors, alike.

Flowcharts on handling common publication lapses are present in a section of the website. Seventeen different issues pertaining to misconduct are available. They provide solutions to problems such as redundant publication, plagiarism, fabrications of data, authorship problems, undisclosed conflict of interest, complaints against authors and reviewers and various types of ethical indiscretions.

The section on guidelines has the code of conduct for its member editors, which encourages them to work for improving the journal by ensuring adherence to quality, freedom of expression, maintenance of integrity and that of adopting a stance of not letting profits come in the way of achieving high intellectual standards. The code also advises the members to meet the needs of the authors and readers and to publish corrections, retractions and apologies, if required.

The research section has details of the work carried out at different places in terms of publication misconduct by grants provided by COPE. Projects on detecting plagiarism, author’s awareness about publication ethics have been successfully completed.

The Forum is perhaps the most interesting section of the website. It hosts all Annual Reports between 1998 and 2008. These reports provide a summary of individual cases linked to the actual description of problem, the advice by COPE, follow-up by the journal and the resolution. The Cases section contains an indexed version of all cases submitted to COPE. These reports and cases should be essential reading for all aspiring authors and those interested in implementing good publication practices.

The Seminar section lists the content from annual seminars organized by COPE. A perusal of the contents reveals a number of interesting cases; the 2010 seminars on plagiarism in the electronic age, cultural differences in plagiarism and screening for plagiarism using a web-based service are remarkable eye-openers.

The publication ethics blog allows members to post comments and to engage in discussion on varied relevant topics. The newsletter of COPE called ‘Ethical Editing’ is also available for download. In addition to news and reports of meetings, it has general articles which tend to demystify some of the more daunting aspects of publication ethics.

The contents of the website are not for the newbie, there is a distance education course in the offing but its details are not available. The problem of misconduct in scientific publication in the developing countries as a unique identity does not find a place on the website. Indian journal members are primarily those from large publishing houses. Smaller Indian journals have not been represented despite a discounted membership rate.

The overall accent of the website is to create awareness about publication ethics and it succeeds in doing so by its content. However, most of the text is uninspiring and only a diehard editor will go through the website in a concerted effort to find solutions. A more pleasing layout with visual appeal and ease of navigation would be more appropriate for such a topical website.

